# Going Electronic: Venturing Into Electronic Monitoring Systems to Increase Hand Hygiene Compliance in Philippine Healthcare

**DOI:** 10.3389/fphar.2022.843683

**Published:** 2022-02-17

**Authors:** Hazel Chloe Villalobos Barbon, Jamie Ledesma Fermin, Shaira Limson Kee, Myles Joshua Toledo Tan, Nouar AlDahoul, Hezerul Abdul Karim

**Affiliations:** ^1^ Division of Biological Sciences, University of the Philippines Visayas, Miagao, Philippines; ^2^ Department of Electronics Engineering, University of St. La Salle, Bacolod, Philippines; ^3^ Department of Natural Sciences, University of St. La Salle, Bacolod, Philippines; ^4^ Department of Chemical Engineering, University of St. La Salle, Bacolod, Philippines; ^5^ Faculty of Engineering, Multimedia University, Cyberjaya, Malaysia

**Keywords:** healthcare-acquired infections, Philippines, hand hygiene compliance, electronic monitoring systems, low-middle-income countries, hand hygiene, monitoring system, infection

## Introduction

Despite the many breakthroughs in the field of public health in the past century, the incidence of healthcare-associated infections (HCAI) remains to be at a high level in both low-middle-income- and high-income-countries across the globe as evidenced by the report from the World Health Organization (WHO) in 2011. Specifically, the Philippines, a low-middle-income country (LMIC), experiences this as well. Despite this, only a few local studies have demonstrated evidence regarding the prevalence of HCAIs, including Navoa-Ng et al. (2011) that reported the prevalence of device-associated infections (DAI), with ventilator-associated pneumonia (VAP) as the most common. The high statistics has also been attributed to a higher mortality rate, especially among adults confined in intensive care units (ICU) (Navoa-Ng et al., 2011).

A way to prevent transmission is through the practice of hand hygiene (HH). It is an integral part of healthcare and is one of the most effective and least expensive methods in preventing the spread of HCAIs (Saharman et al., 2019). Despite this, HH compliance remains to be low in many countries, both in LMICs and high-income countries (HICs) (WHO, 2013). In an effort to increase compliance, the WHO developed approaches seeking to improve HH behavior but is needing additional improvements. Optimization of these approaches may be applied through the use of electronic monitoring systems (EMS), a solution that may also be applied in the Philippines.

### Prevalence and Impact of HCAIs in LMICs Especially the Philippines

Healthcare-associated infection (HCAI), as defined by the WHO (2011), is an infection introduced to a patient during the process of treatment in healthcare facilities. This type of infection can appear during or after the patient receives treatment. Studies have shown that the prevalence of HCAIs has been consistently high in many countries, with much literature (Cai et al., 2017; Cassini et al., 2016; US CDC, 2019.^1^) focused on reporting about HICs (WHO, 2011).

Fewer studies give a depiction of the rates in LMICs, but findings report that the rate of HCAIs is significantly elevated as well (WHO, 2011). Results of the meta-analysis and systematic review conducted by Allegranzi et al. (2011) reports that one of the major burdens for patients and healthcare workers (HCWs) in LMICs, is the prevalence of HCAIs affecting as much as 15.5 per 100 population.

Limited studies investigate the rates of HCAIs in LMICs and even more so in the Philippines. The study of Navoa-Ng et al. (2011) conducted in the ICUs of only 3 hospitals in the country reported 183 DAIs in 2005–2009. Further reporting that ventilator-associated pneumonia (VAP), constituting 67.2% of the cases, as the most common DAI in adult ICUs. This was followed by catheter-associated urinary tract infections (CAUTI) and central line-associated bloodstream infections (CLABSI) at 22.4 and 10.4%, respectively.

HCAIs have been found to negatively impact many healthcare systems in LMICs, causing patients to increase their length of stay in the ICUs and have caused serious cases of morbidity and increased the rate of mortality (Navoa-Ng et al., 2007; Navoa-Ng et al., 2011). Such impacts can be mitigated by decreasing HCAI rates through behavioral change by HCWs, one of which can be done through increasing HH compliance (WHO, 2011).

### Approaches to Increase HH Practices and its Applicability in LMICs

One of the most common ways of transmitting HCAIs is through the hands of the HCWs (Pittet et al., 2006). As such, it is vital that proper HH is practiced among HCWs as a preventive measure. Hand hygiene has been proven to be effective at preventing transmission of all types of HCAIs. However, despite its simplicity and effectiveness, compliance to HH practices remains to be low in many countries, reportedly only 40% in HICs, with LMICs reported to have much lower statistics (Erasmus et al., 2010; Phan et al., 2018).

The WHO, seeking to standardize HH practices, developed “*My 5 Moments of Hand Hygiene*” (MFMHH). This method is based on the concept of finding optimal points during healthcare delivery to perform HH practices in order to disrupt the microbiological transmission of pathogens. The moments identified using this concept include: 1) before contact with patient; 2) before performing aseptic task; 3) after exposure to patient’s body fluids; 4) after contact with patient; and 5) after contact with patient’s surroundings (WHO, 2009b).

This method has been largely aimed at educating HCW, as well as in reporting their HH practices. Its application in LMICs, including the Philippines, may be hindered because it involves dividing the healthcare environment into two zones: the patient zone, which includes the patient and the space that surrounds them, and the healthcare zone, which includes all the other surfaces in the area not classified under the former. Many healthcare facilities in LMICs, including the Philippines, have overcrowding problems making the division of environment non-feasible (Loftus et al., 2019). Averaging at only 10.7 beds per 10,000 population, the Philippine health care facilities bed capacity is well below the WHO recommendation of 20 beds per 10,000 population (Alliance for Improving Health Outcomes Inc., 2017).^2^ In comparison, HICs average at around 53 hospital beds per 10,000 population (The World Bank Group, 2021).^3^

The patient zone includes the patient and the immediate space around them. Furthermore, anything that is frequently touched by the patient such as their bed railing, bed linen, etc., as well as anything frequently touched by HCWs when they are providing care to the patient are also classified as belonging to the patient zone. These are areas that are often contaminated by the patient but is cleaned after every patient admission. Healthcare zone, on the other hand, includes all other areas not included in the former, including other patients’ zone and other areas in the healthcare facility. These are areas where foreign microorganisms for a particular patient that can potentially infect them are present (Malik, 2008). Many healthcare facilities in LMICs, including the Philippines, have overcrowding problems making the division of environment non-feasible (Loftus et al., 2019; Lowe et al., 2021; Tyagi et al., 2018). Averaging at only 10.7 beds per 10,000 population, the Philippine health care facilities bed capacity is well below the WHO recommendation of 20 beds per 10,000 population (Alliance for Improving Health Outcomes Inc., 2017). In comparison, HICs average at around 53 hospital beds per 10,000 population (The World Bank Group, 2021).

Apart from MFMHH, the WHO also pushed for the use of a multi-modal approach in increasing compliance to HH practices. This approach involves five components: system change, training and education, evaluation and feedback, reminders in the workplace, and institutional safety climate (WHO, 2009a; Pfäfflin., 2017; Müller et al., 2021).

This involves the participation of the administration in crafting policies and guidelines and conducting educational and training campaigns to better encourage healthcare workers to engage in HH practices. Being multi-modal, this approach gives more room for tailoring interventions into the context of a healthcare facility.

These approaches can serve as an overarching intervention to increase HH compliance. To gauge progress, monitoring systems must be implemented.

### Monitoring Systems

Effective HH monitoring systems in healthcare facilities are needed to know HH compliance by HCWs. A traditionally used method and the gold standard is direct observation (WHO, 2009b). The WHO developed a way to quantify HH compliance based on MFMHH. This method involves a trained observer who is tasked to monitor the HH behavior of HCWs. Although proven to be effective and simple enough to be implemented in LMICs, this method presents some limitations. One of these includes being prone to biases, especially observer’s and selection biases. Besides this, the method can be time-consuming and need a number of employed observers to accurately describe the HH practice in a big healthcare facility (Masroor et al., 2017).

A way to remove these biases is to employ an EMS, a method in HH monitoring that has just recently been explored. EMS eliminates the need to employ a trained observer and is not prone to the aforementioned biases. Furthermore, EMS is more cost-effective in the long run as it can provide continuous real-time monitoring and feedback even in large facilities (Masroor et al., 2017). There are two types of EMS that will be explored in this paper.

### Electronic Monitoring Systems

There are a variety of EMS types that have been developed in recent years. This paper will focus on technologies utilizing real-time locating systems, namely, *MediHandTrace* and *Biovigil*. Both systems primarily utilize radio frequency identification (RFID) (Boudjema et al., 2013; McCalla et al., 2017).

**FIGURE 1:**
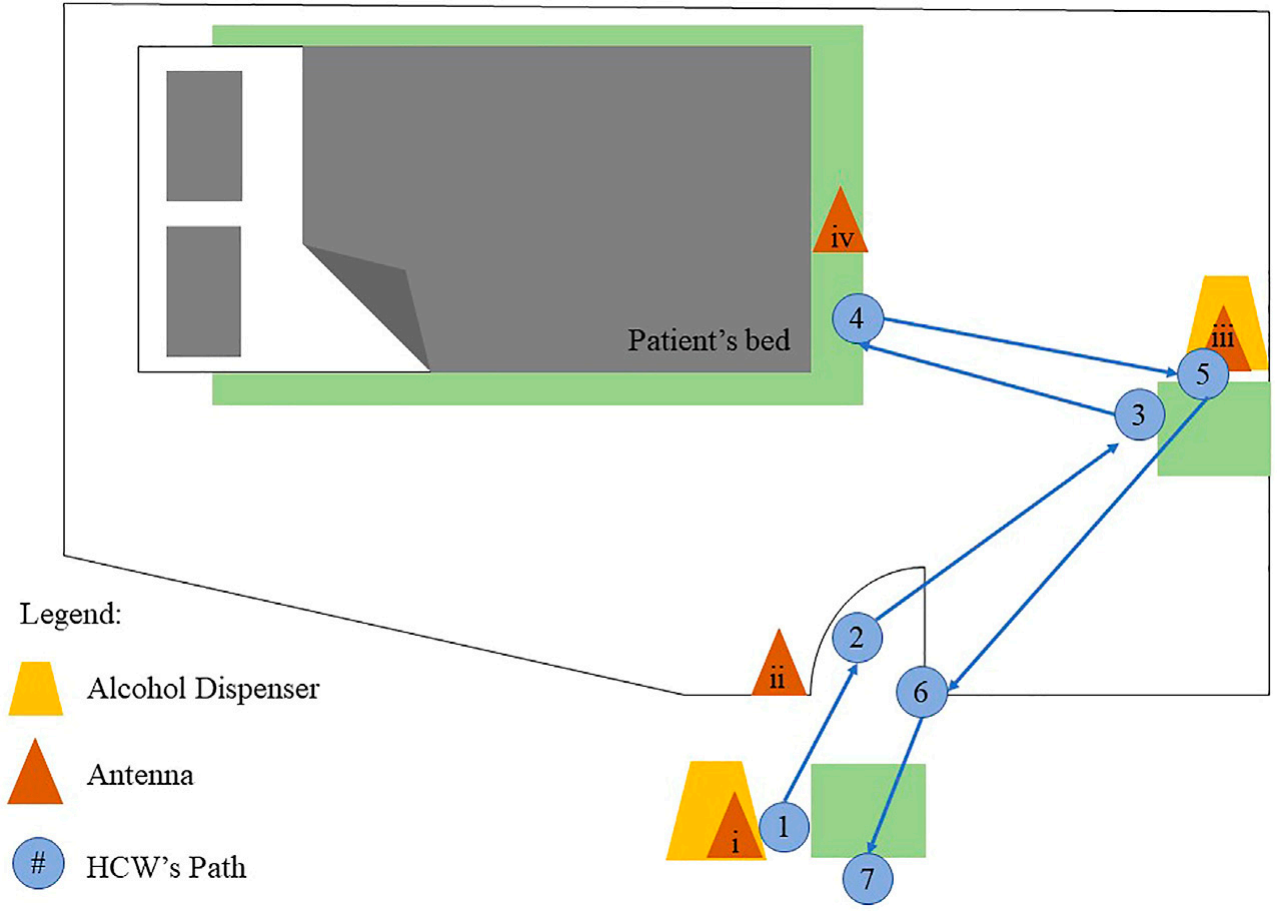
Antennae placement within patient room (adopted from Boudjema et al., 2013).

For MediHandTrace, a specific RFID technology, “iCode RFID 15693” tag technology, is used together with an alcohol dispenser motor. In the pilot study conducted by Boudjema et al. (2013), four antennas were placed within the room, placed strategically to monitor HH based on MFMHH. The antennas were placed in the following locations: 1) outside the room under an alcohol dispenser; 2) door entrance; 3) inside the room under another alcohol dispenser; and 4) around the bed within a secured zone. A tag is inserted within the shoe of an HCW to determine their location within the room. Sensors were also placed near the two alcohol dispensers to measure the number of sprays as well as the volume used at a time. The placement of the antennae in the patient room is visualized in Figure 1. Data from the antenna, tag, and sensors were then read by another technology, which then transfers the data into the main server through an Ethernet connection. In the study, verification of whether the technology accomplished its purpose was verified through video cameras stationed within the room for the course of the study (Boudjema et al., 2013).

AADO modifies the traditional pen and paper method of recording observations by enabling observers to record their findings on mobile or tablet applications (Wiemken et al., 2018). These methods still employ direct observers but there is a marked reduction in the time spent on the transcription of data, as well as decrease the risk of introduction of errors in the analysis. (Cawthorne et al., 2021; Wang et al., 2021). Despite its potential, AADO technology is met with numerous challenge and limitations, the most prominent of which is its non-user-friendly nature and subsequent intensive training needed to be done for HCWs (Cawthorne et al., 2021).

CAO utilizes cameras either situated inside patient rooms or mounted in HCW’s chests that monitors HH practices that are then analyzed by either human observers or an algorithm (Brotfain et al., 2017; Wang et al., 2021). An example of this technology was used in the study by Awwad et al. (2018) which utilized positioned cameras in a patient’s room to capture images which were then analyzed using computer vision techniques to detect HH practices in accordance with the WHO’s MFMHH. However, this technique faces ethical issues as cameras can record not only HH practices but also patients during treatment (Gon et al., 2020a).

SAO employs the use of sensors, placed in either electronic dispensers, electronic dispensers combined with other sensors, and inertial measurement unit and microphone (IMU). In essence, electronic dispenser technology detects the usage frequency and dispense of HH products in patient’s rooms. IMU, on the other hand, detects and measures HCW’s movements which are then analyzed to determine their HH practices (Wang et al., 2021). This technology offers some advantages over direct observation including lesser expense as it no longer involves employing human observers, however it lacks the depth of analysis in the quality of HH practices performed by HCWs (Kato et al., 2021).

RLTS measures HH practice by sensing dispenser use and HCW movements inside a patient’s room by utilizing either of the following: radio frequency identification (RFID), infrared, Bluetooth, ultrasound, and Wi-Fi (Wang et al., 2021).

In this paper, the focus will be on technologies utilizing real-time locating systems, specifically those that primarily utilize RFID. RFID tags are commonly placed within a HCW’s clothing or are worn as bracelets or wristbands and are used in combination with sensors situated within the room to detect HCW compliance to HH practice. This information is then collected and communicated to a cloud server located in the hospital (Tarantini, 2019; Periyasamy et al., 2020; Huang et al., 2021; Wang et al., 2021).

Three systems utilizing RFID technology will be discussed in the following sections, MediHandTrace, and Biovigil (Boudjema et al., 2013; McCalla et al., 2017).

In contrast, Biovigil, although also utilizing RFID, functions with the use of a wristband that shows different colors indicating whether the HCW has performed a HH practice. These bands show a yellow-colored light which progresses to red to prompt the HCW to engage in HH practice. Once HH is done, the band will show a green-colored light, indicating that HH has been adequate. The band can also produce auditory prompts to signal HCWs to perform HH. Sensors within these bands can detect whether the HCW used soap and water or alcohol-based rub to sanitize their hand. After an HCW’s shift, data is gathered while the band is charging at a base station; this is then transmitted to cloud-based storage where it can be analyzed (McCalla et al., 2017).

## Discussion

The WHO has provided several guidelines to monitor HH in healthcare facilities. The first step in increasing HH compliance would be to create interventions that can make the environment in healthcare facilities more conducive to allow practice of MFMHH. This might be a lengthy process, especially in LMICs, and will require a sizable investment of resources and commitment from the hospital and government administration. Furthermore, the government must allocate more resources focused on the surveillance of HCAIs in the country since there is an evident lack of recent data reports and literature reporting on the prevalent types of HCAIs, at the national level. Once this is achieved, context-specific interventions and an optimized use of EMS will be possible and can aid in increasing HH compliance in healthcare facilities.

Given the problem of overcrowding in LMICs, making it harder to differentiate spaces to allow practice of MFMHH, incorporating an EMS that works similarly with MediHandTrace will be difficult. In the Philippines, where there is an insufficient number of beds for the population, EMS working similarly to MediHandTrace will most likely be unsustainable and difficult to implement. The study conducted by Dufour et al. (2017) utilizing this technology even had mixed results, concluding that HH compliance is still HCW-dependent. This study was conducted in France, a HIC, which has a better hospital bed to population ratio. Given its less than passable results coupled with its requirement of several system changes to work, applying it to a LMIC like the Philippines may not be a cost-effective decision.

In contrast, Biovigil, an EMS utilizing a wearable technology that can prompt a HCW to perform HH practice, may be more suitable in the Philippine context. Based on the information from study of McCalla et al. (2017), this EMS does not require many changes in the system. As such, EMS using a technology like Biovigil is a better recommendation since the Philippines has not yet been able to resolve the problems of patient overcrowding and does not appear to be able to solve it in the near future.

The application of a RFID system in a hospital setting as seen in Mitchell et al. (2017) is evidence that utilizing this type of technology is possible in the Philippines. With their study providing proof that such technology provides benefits in improving individual HCW HH practice, it is impartial to suggest that this technology will indeed be helpful in the improvement of HH practice. More studies in the future can follow and investigate which type of EMS is best to use given a certain hospital’s context. Perhaps, a large amount of research, as well as the overall improvement of public hospitals in the country is needed before this can be achieved. However, the point still stands that there is a great need to improve on the monitoring systems in the hospitals in the country and starting the application of EMS in hospitals is an excellent step towards that goal.

All in all, it is important that any technology used as EMS can be incorporated seamlessly into the healthcare system of the country. Based on the multi-modal approach of the WHO, to address HH compliance to lower HCAIs, the healthcare system must first be improved. To do this, incorporating these EMS will soon follow. Given the established fact that HH is an effective way to combat HCAIs like DAIs including VAP, CAUTI, and CLABSI, increasing HH compliance through EMS can greatly decrease the transmission rate of the aforementioned infections. As such, the impact of EMS lies in its ability to increase HH compliance in healthcare facilities. When this is done, the incidence of HCAIs will decrease.

## Footnotes

1
https://www.cdc.gov/hai/data/portal/progress-report.html

2
https://www.aiho.org.ph/2017/05/08/philippine-health-system/

3
https://data.worldbank.org/indicator/SH.MED.BEDS.ZS?end=2019&start=1960&view=chart
